# Assessment of Perceived Physical Literacy and Its Relationship with 24-Hour Movement Guidelines in Adolescents: The ENERGYCO Study

**DOI:** 10.3390/ijerph22020194

**Published:** 2025-01-29

**Authors:** Víctor Manuel Valle-Muñoz, Estela Águila-Lara, Manuel Ávila-García, José Manuel Segura-Díaz, Pablo Campos-Garzón, Yaira Barranco-Ruiz, Romina Gisele Saucedo-Araujo, Emilio Villa-González

**Affiliations:** 1 Department of Physical Education and Sports, Faculty of Sport Sciences, Sport and Health University Research Institute (iMUDS), University of Granada, 18071 Granada, Spain; victor_96@go.ugr.es (V.M.V.-M.); estelagl@correo.ugr.es (E.Á.-L.); ybarranco@ugr.es (Y.B.-R.); evilla@ugr.es (E.V.-G.); 2 “La Inmaculada” Teacher Training Centre, University of Granada, 18013 Granada, Spain; 3 Faculty of Sport Sciences, University Isabel I, 09003 Burgos, Spain; 4 Department of Physical and Sports Education, Faculty of Education and Sport Sciences, Sport and Health University Research Institute (iMUDS), University of Granada, 52005 Melilla, Spain; jmsegdia@ugr.es; 5 Department of Public Health, Faculty of Health Sciences, University of Lethbridge, Lethbridge, AB T1K 3M4, Canada; pcampos@ugr.es; 6 Department of Global Public Health, Karolinska Institutet, 171 76 Stockholm, Sweden; 7 Department of Specific Didactics, Faculty of Education, University of La Laguna, 38200 San Cristóbal de La Laguna, Santa Cruz de Tenerife, Spain; rsaucedo@ull.edu.es; 8 Sport and Health University Research Institute (iMUDS), University of Granada, 18071 Granada, Spain

**Keywords:** physical literacy, physical activity, adolescents, screen time, movement behavior

## Abstract

Scientific evidence suggests that meeting the 24-hour movement guidelines is associated with numerous health benefits. One concept that emphasizes an individual’s active lifestyle is physical literacy (PL). However, the relationship between PL and 24 h movement guidelines in adolescents has not been analyzed to date. The aim of this study was to examine the relationship between perceived physical literacy (PPL) and adherence to the 24-hour movement guidelines in Spanish adolescents. This cross-sectional study included a total of 56 adolescents (mean age 13.2 ± 1.3 years). PL was assessed using the Spanish Perceived Physical Literacy Instrument for Adolescents (S-PPLI), categorizing participants into low, medium, and high PPL levels. To determine compliance with movement guidelines (24-hour movement), physical activity was assessed through accelerometry, while screen time and sleep duration were evaluated using questionnaires. One-way ANOVA and chi-square analysis were used to examine PPL levels and adherence to the 24-hour movement guidelines. The results indicated that higher levels of PPL were associated with greater adherence to the 24-hour movement guidelines. Specifically, most participants met one or two recommendations as PPL increased (*p* = 0.002). In the medium PPL group, 50% met one recommendation, 37.5% met two, and 12.5% did not meet any, while in the high PPL group, 52.8% met one recommendation, 41.7% met two, and 5.6% did not meet any. These findings suggest that higher levels of PPL are associated with greater adherence to the 24-hour movement guidelines. Future studies should explore this association in larger samples of Spanish adolescents and should promote the practical implications regarding the importance of designing educational programs. This should be achieved through curricula that are based on PL and that integrate strategies to reduce screen time, promote healthy sleep habits, and foster a comprehensive and sustainable understanding of these among adolescents.

## 1. Introduction

Adequate sleep, regular physical activity (PA), and reduced sedentary behavior, such as by limiting screen time, are widely recognized as key factors for healthy development during childhood and adolescence [1,2,3]. While often considered independently, these modifiable lifestyle factors are interconnected and form a continuum of movement-related behaviors that influence one another [4]. These guidelines emphasize that the combination and interactions among all components of 24-hour movement should aim to maximize health benefits [4]. Despite this, a recent systematic review and meta-analysis of 387,437 youth from 23 countries conducted by Tapia-Serrano et al. [5] reported that overall adherence to 24-hour movement guidelines is only 7.12% among adolescents. In response to these findings, the Canadian 24-hour movement guidelines were published in 2020. These guidelines recommended that youth engage in ≥60 min of moderate to vigorous physical activity (MVPA) per day, sleep 9–11 h daily (for children aged 5–13 years) or 8–10 h daily (for adolescents aged 14–17 years), and limit recreational screen time to no more than 2 h per day [1]. Supporting these guidelines, recent studies have found positive associations between the number of guidelines met and overall health indicators in children and adolescents [6]. Participants meeting more components of the guidelines showed lower levels of adiposity [7], better health-related quality of life [8], higher psychosocial scores [9], reduced risk of depression [10], improved academic performance [11], and higher global cognition scores [12]. Research consistently demonstrates that the combination of sufficient PA, adequate sleep patterns, and reduced screen time during this formative stage yields numerous positive health outcomes across physical, social, mental, and cognitive domains, laying the foundation for lifelong health [13,14]. Adolescence is recognized as a crucial phase for developing autonomy and establishing long-term health behavior patterns that persist into later stages of life [15]. Inadequate physical activity development during the transition from childhood to early adulthood can contribute to more significant health issues among emerging adults [15]. Despite its critical importance, this stage of life tends to be overlooked in health promotion initiatives, underscoring the urgent need for greater scientific attention to sustaining lifelong active lifestyles [16].

In this context, a novel concept that has recently gained attention for promoting a healthy and active lifestyle throughout life is physical literacy [17]. The International Physical Literacy Association (IPLA) defines PL as “the motivation, confidence, physical competence, knowledge, and understanding to value and take responsibility for engaging in physical activities throughout life” [18]. As a holistic and multidimensional concept, PL provides individuals with affective, cognitive, physical, and behavioral attributes that could be integrated into the capacity for a physically active lifestyle [19]. This means that it involves not only components of the physical domain (e.g., fundamental movement skills) but also components related to the cognitive domain (e.g., knowledge and understanding) and the affective domain (e.g., motivation and self-efficacy) [20].

Empirical evidence has shown that physically literate individuals engage in higher levels of PA, achieve optimal sleep duration, and exhibit lower levels of sedentary behavior, as assessed through measurement tools such as PLAYfun, PLAYparent, PLAYself, and the Canadian Assessment of Physical Literacy (CAPL) [21,22,23]. Additionally, other studies conducted on Spanish adolescents showed that high levels of PL are associated with higher levels of muscular fitness and strengthening activities [24], as well as with better academic performance [25] and a lower likelihood of being overweight or obese [26]. Moreover, PL is positively associated with the emotional and social well-being of adolescents. A study on adolescents around 12 years old revealed that PL was positively correlated with participation in physical education, leisure-time exercise, positive affect, and vitality, while it was negatively correlated with negative affect [27]. Furthermore, PL could reduce potential mental health issues among adolescents [28], contributing to the maintenance of habits in later stages of life [21].

To date, only one study has examined the relationship between PL scores in children aged 8 to 12 years and the Canadian 24-hour Movement Guidelines. This study found that children who met the recommendations for PA or sedentary behavior scored higher in the PL domains of physical competence, motivation, and confidence compared to those who did not adhere to any of the guidelines. Adherence to the 24-hour Movement Guidelines was assessed through self-reported questionnaires and the use of a pedometer to record daily step counts, while the CAPL was used to estimate total PL [29]. However, the relationship between PL and the 24-hour movement guidelines remains an underexplored area, particularly when behaviors are assessed using objective tools such as accelerometry that are combined with self-reported questionnaires. In the specific case of Spanish adolescents aged 12 and older, this gap is especially relevant. Adolescence is a critical developmental stage during which movement patterns, behavioral preferences, and attitudes toward PA are constantly evolving. Furthermore, social and technological changes, such as increased screen time and alterations in sleep patterns, make it essential to understand how PL levels relate to adherence to integrated movement guidelines within this cultural context. A thorough and culturally contextualized analysis of this relationship would allow for the design of more tailored and effective interventions aimed at improving both physical health and general well-being. Therefore, addressing this gap in the literature is crucial to understanding how PL impacts the integration of 24-hour movement behaviors. This holistic approach enables the analysis of the interaction between all movement behaviors, rather than focusing solely on isolated components. It is anticipated that adolescents with higher levels of PL will not only demonstrate greater participation in MVPA activities but will also be less likely to engage in recreational screen use and will exhibit healthier sleep patterns. The significance of this approach lies in its potential to inform educational and public health policies that promote PL as a key tool for fostering sustainable lifestyles.

Therefore, future studies should investigate the causal relation between PPL and adherence to movement guidelines, providing a comprehensive understanding of these behaviors and their influence on a healthy lifestyle.

## 2. Materials and Methods

### 2.1. Study Design and Participants

The present study is based on a cross-sectional and descriptive design derived from the ENERGYCO project (ClinicalTrials.gov: NCT06414668), which was approved by the Human Research Review Committee of the University of Granada (Reference: 2496/CEIH/2021). This project consists of two phases, and specifically, the data for this study correspond to the first phase of the project, conducted between October 2023 and March 2024. The recruitment of the project participants was based on dissemination in social networks and contact via email and telephone with secondary schools in the provinces of Granada, Almería and Jaén. Specifically, for the present study, a subsample of 56 adolescents (13.2 ± 1.2 years, 40 males and 26 females) was selected by convenience, meeting the following inclusion criteria: (1) they provided informed consent signed by their parents at the beginning of the study; (2) they were within the required age range (12–18 years); and (3) they did not present any medical condition limiting physical activity or requiring special care.

### 2.2. Instruments

#### 2.2.1. Anthropometry

The children’s height and body mass measurements were taken to calculate body mass index (BMI), with all adolescents wearing shorts, a short-sleeved shirt, and bare feet for both measurements. Body mass was measured with a 0.1 kg approximation using a Seca 876 weighing system (Seca, Ltd., Hamburg, Germany). Height was measured in the Frankfort plane with an approximation of 0.1 cm using a Seca 213 stadiometer (Seca, Ltd., Hamburg, Germany). Height and body mass were measured twice, with the averages taken for both measurements. BMI was calculated as body mass in kilograms divided by height in meters squared.

#### 2.2.2. Perceived Physical Literacy

Perceived physical literacy (PPL) was assessed using the PPL instrument for adolescents [30] in its Spanish adaptation as S-PPLI, which is a valid and reliable instrument for measuring PPL among Spanish adolescents [31] (Cronbach’s α = 0.87). This instrument consists of 9 items scored on a 5-point Likert scale, from 1 (strongly disagree) to 5 (strongly agree), grouped into 3 factors (3 items each): knowledge and understanding; self-expression and communication with others; and sense of self and self-confidence. To calculate the total score, the scores of all items are added together, resulting in a value that can range from a minimum of 9 to a maximum of 45 points. Based on this total score, individuals were classified into three categories: low PPL (9–31 points), moderate PPL (32–36 points), and high PPL (37–45 points) [24].

#### 2.2.3. Canadian 24-Hour Movement Guidelines

The Canadian 24-hour movement guidelines are a combination of three key health indicators in children and adolescents aged 5–17 years focusing on adherence to daily guidelines for PA (at least 60 min of MVPA per day), sleep time (9–11 h of uninterrupted sleep per day) and screen time (less than 2 h per day) [1,32]. To analyze compliance with these recommendations, participants were classified into four categories: not meeting guidelines; meeting exclusively one guideline; meeting exclusively two guidelines, and meeting three guidelines. The methodology and instruments used to assess the variables of PA, sleep time duration and screen time are described below.

#### 2.2.4. Physical Activity

PA time was measured using a triaxial accelerometer (Actigraph Wgt3x-BT, Pensacola, FL, USA). This instrument records acceleration in the three axes of movement, reporting the amount of activity performed, and is a valid and reliable tool to objectively measure physical activity in adolescents [33]. The research team attached the accelerometer to each participant’s right waist through an elastic belt and instructed them on how to take care of the accelerometers. The participants had to wear their accelerometer for 24 h a day, 7 days a week, except during bathing, water activities, and sleeping hours.

Accelerometer data were processed in the open-source R package GGIR v.3.1-2 [34]. The GGIR pipeline included (i) automatic calibration of raw accelerometer data to local gravitational acceleration, (ii) detection and imputation of idle time, and (iii) calculation of activity counts, which were processed at a sampling rate of 90 Hz and set to record in 15 epochs using the algorithm described by Neishabouri et al. [35]. The cut-off point to determinate moderate-to-vigorous intensity was 574 counts/15 s [36].

#### 2.2.5. Sleep Time Duration

The participants’ sleep duration was assessed using an ad-hoc document with a series of questions indicating their usual sleep onset time and usual wake-up time during school and non-school days. Participants who reported a sleep duration within the recommended range (8 to 10 h/night for children aged 14 to 17 years) were considered to meet the sleep duration recommendation [1]. Those who reported insufficient sleep were considered not to meet the sleep duration recommendation.

#### 2.2.6. Screen Time

Sedentary screen time was measured using the Youth Activity Profile Spanish Version (YAP-S) questionnaire [37] (kappa coefficients = 0.57). This questionnaire analyses PA performed during and outside school as well as sedentary habits related to screen time [37]. In relation to screen time, participants were asked about the use of: ‘time watching TV’, ‘playing video games’, ‘using a computer’ and ‘mobile phone time’. For each type of screen, participants were asked to tick one of the following response options: (1) No screen time, (2) less than 1 h per day, (3) between 1 and 2 h per day, (4) more than 2 h and up to 3 h per day, or (5) more than 3 h per day.

To measure total screen time, total screen time was calculated by summing the responses for the four types of screen time (‘watching TV’, ‘playing video games’, ‘using a computer’ and ‘mobile phone time’). For example, if a participant responded ‘less than 1 h per day’ for all categories, the total would be ≤4 h/day. Finally, participants who reported more than 2 h and up to 3 h/day were classified as non-compliant with the recommendations. In addition to calculating the total screen time, which was determined by summing the daily hours of screen time (TV, video games, computer, and mobile phone) for the study subject, the cut-off point for meeting the guidelines was set at 4 h (inclusive).

### 2.3. Data Analyses

Descriptive statistics were calculated for all measured variables. The Kolmogorov–Smirnov test was used to examine the normality of all variables. To compare PPL (low, medium, high) in relation to quantitative variables (minutes of MVPA and sleep time duration), a one-way ANOVA test was performed. The homogeneity of variances was analyzed using Levene’s test. Post hoc tests were conducted using the Bonferroni statistic to identify which behaviors were significantly different from each other. However, for the qualitative variable (screen time), the comparison was performed using Pearson’s chi-square analysis. In addition, chi-square analyses were performed to estimate the relationship between the number of met recommendations (PA, sleep time and screen time) and the level of PPL (low, medium, high). All statistical analyses were performed with SPSS version 28.0 for Windows (IBM), and the level of significance was set at *p* < 0.05.

## 3. Results

The descriptive data for the total sample (n = 56), broken down by each PPL category (low, medium, and high), are presented in Table 1. Study participants ranged in age from 12 to 16 years (13.2 ± 1.2 years). The proportion of adolescents with high PPL scores (n = 41, 73.2%) was considerably higher compared to those in the medium (n = 9, 16.07%) and low PPL groups (n = 6, 10.7%). In terms of sex, males represented 72.7% of the total participants and were predominantly in the high PPL group, whereas females were more represented in the low PPL group (60% of the low PPL group compared to 22% of the high PPL group). Additionally, adolescents in the low PPL group had a higher BMI (19.8 ± 2.7 kg/m^2^) compared to those in the medium and high PPL groups, with an average difference of 1.1 kg/m^2^ between the low and high PPL groups.

Regarding movement behaviors, the results indicated that there was no significant relationship between PPL status and the variables of PA, sleep, and screen time. Regarding MVPA, the average time of MVPA was lowest in the low PPL group (45.82 min/day), while the medium and high PPL groups had higher MVPA levels (77.17 ± 33.94 min/day and 72.56 ± 40.45 min/day, respectively). However, the differences between the groups were not statistically significant (F = 0.285, *p* = 0.754, *η*^2^ = 0.01). Regarding sleep time, the medium and high PPL groups showed similar averages (9.25 ± 0.87 h/day and 9.17 ± 0.85 h/day, respectively), while the low PPL group had a slightly lower average time (8.60 ± 0.97 h/day). Although a trend toward longer sleep durations was observed at higher PPL levels, these differences were not significant (F = 1.082, *p* = 0.346, *η*^2^ = 0.03). The average sleep duration was lower in the low PPL group (8.6 ± 0.9 h/day), while the medium and high PPL groups showed higher sleep levels (9.2 ± 0.8 h/day and 9.1 ± 0.8 h/day, respectively). As for screen time, it was identified that mobile phones were the most used device across all groups, with an average of 3.6 h/day. Adolescents with low PPL showed a higher average usage time (4.8 ± 0.4 h/day) compared to those in the medium and high PPL groups (3.4 ± 1.1 h/day and 3.6 ± 1.1 h/day). However, the total screen time was higher among adolescents with low PPL (11.5 ± 1.0 h/day) compared to those in the medium and high PPL groups (10.3 ± 3.5 h/day and 9.6 ± 2.5 h/day). While the descriptive differences in screen time between the low, medium, and high PPL groups suggest a trend toward greater screen time in adolescents with low PPL, no statistically significant differences were found between the groups (χ^2^ = 27.890, gl = 22, *p* = 0.179).

**Figure 1:**
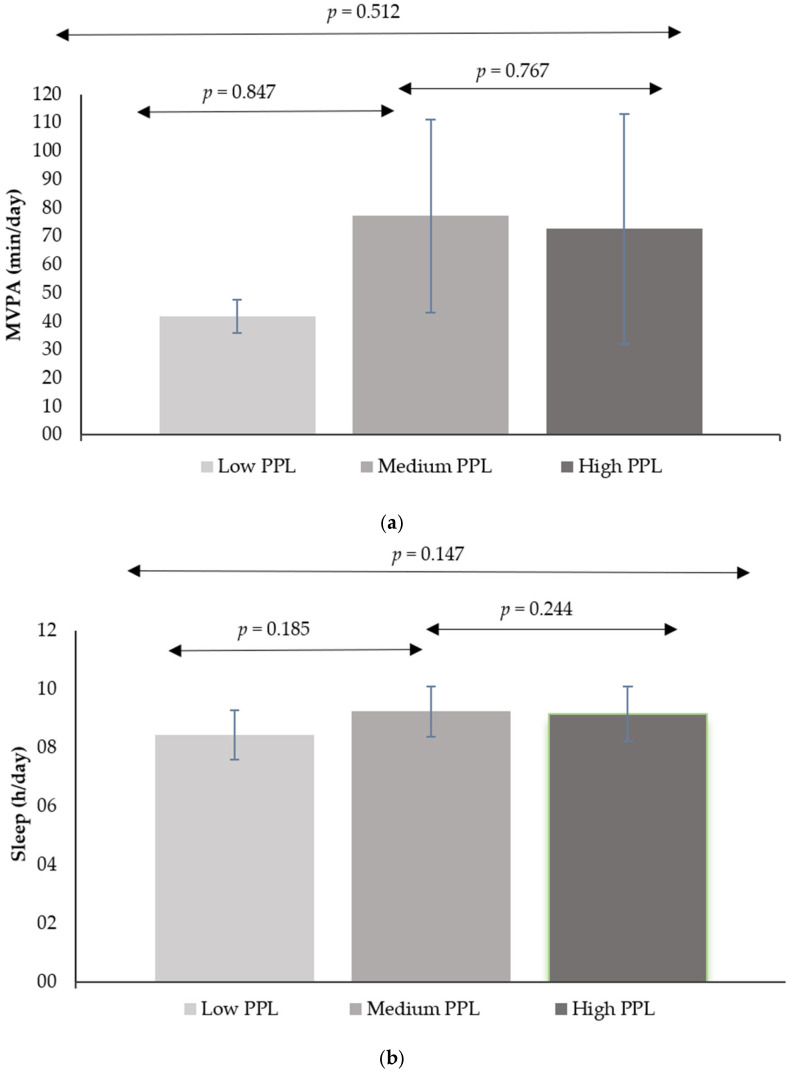
(**a**) Mean predicted MVPA values were categorized by PPL status into one of three categories: Low PPL (9–31 points), medium PPL (32–36 points), and high PPL (37–45 points). (**b**) Mean predicted sleep values were categorized by PPL status into one of three categories: Low PPL (9–31 points), medium PPL (32–36 points), and high PPL (37–45 points).

The results of the marginal means for time spent in MVPA and sleep time, broken down by PPL status, are presented in Figure 1. No statistically significant differences were observed between PPL groups in terms of MVPA time (*p* = 0.512). In this regard, the poc-hoc analyses showed no significance between the groups: Low-medium PPL (*p* = 0.847) 95% CI [−101.51, 39.84] and medium-high (*p* = 0.767) 95% CI [−41.4, 112.25]. Regarding sleep time, no differences were also observed between the PPL groups (*p* = 0.147). Consequently, the post-hoc analyses showed no significance between the groups: Low-medium PPL (*p* = 0.185) 95% CI [−1.65, 0.21] and medium-high (*p* = 0.244) 95% CI [−0.32, 1.92]. Similarly, no significant differences were found in total screen time across the PPL categories (*p* = 0.277).

**Figure 2:**
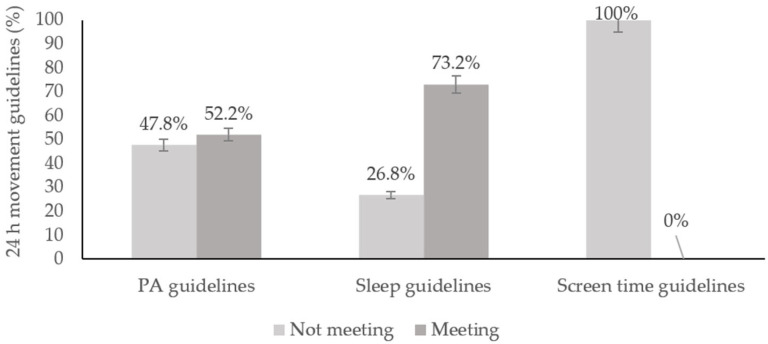
Bar chart showing the proportions (%) of adolescents meeting the Canadian 24-Hour Movement Guideline recommendations: PA, sleep and screen time guidelines (n = 56).

**Figure 3:**
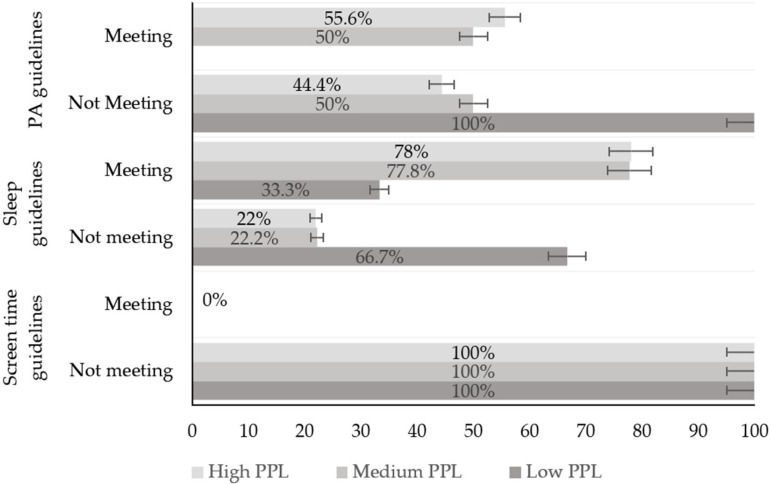
Bar chart showing the proportions (%) of adolescents meeting each of the Canadian 24-Hour Movement Guideline recommendations: PA, sleep and screen time guidelines based on their PPL status.

Figure 2 shows the prevalence of adolescents who met each of the 24-hour movement guidelines. Overall, most participants (73.2%) met the sleep guidelines, while only 52.2% met the PA guidelines. The relationship between adherence to each of the 24-hour movement guidelines and PPL status is presented in Figure 3. The results show that participants with a high PPL level are more likely to meet the PA guidelines, get an adequate sleep duration, and limit screen time compared to those with a low PPL level.

**Figure 4:**
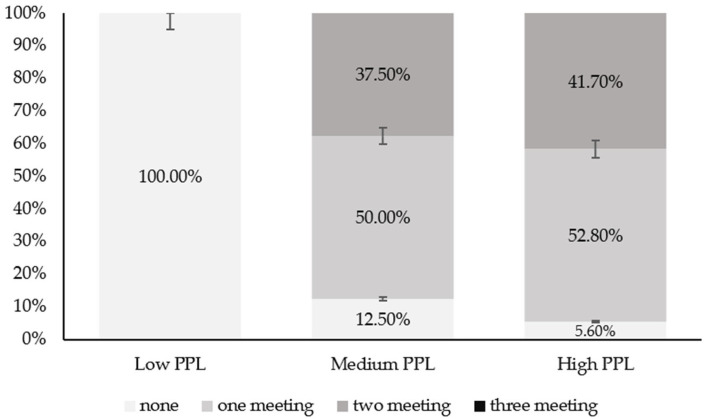
Percentage of adolescents in each category meeting movement recommendations (none, meeting 1 guideline, meeting 2 guidelines or meeting 3 guidelines) within each PPL status.

Figure 4 shows the percentage of adolescents in each adherence category to the movement guidelines within each PPL status. The results indicate a significant relationship (χ^2^ = 10.876, df = 4, *p* = 0.028, *V =* −0.01) between the adherence categories to the 24-hour movement guidelines (meets/does not meet) and the PPL categories (low, medium, and high). In the case of medium PPL, 50.0% met at least one recommendation, compared with 52.8% in the high PPL category. Additionally, 37.5% of participants in the medium PPL category met two recommendations, versus 41.7% of participants in the high PPL category. It is noteworthy that no participants met all three recommendations, regardless of their PPL level. Furthermore, 100% of participants with low PPL did not meet any recommendation.

## 4. Discussion

This study aims to compare PL in relation to MVPA, screen time, and sleep, as well as to examine the relationship between PL and the Canadian 24-hour movement guidelines in Spanish adolescents. Overall, our key findings suggest that adolescents with medium and high PPL levels demonstrate greater adherence to these guidelines. Additionally, higher PPL levels are associated with increased compliance with guidelines. However, despite this positive trend, none of the participants met all three recommendations, primarily due to elevated screen time across all groups. This highlights PPL as a gateway to lifelong PA, potentially shaping 24-hour behavior patterns towards healthier lifestyles [22]. Previous studies have focused on children, with limited attention paid to adolescents. This research emphasizes the importance of fostering PPL in adolescents in order to establish long-term active lifestyles.

Our findings align with previous studies. Belanger et al. [21] evaluated the relationship between PL levels and adherence to PA and sedentary behavior guidelines among Canadian children (n = 2956, 56.6% girls) aged 8–12 years. They found that children scoring high in physical competence and motivation and confidence were more likely to meet the guidelines, with a significant relationship between PL and guideline adherence (*p* < 0.05). Similarly, a cross-sectional study involving Chinese university students (19.2 ± 1.2 years) showed that 36.5% (n = 291) met all three 24-hour movement guidelines, while 4.1% (n = 33) met none. Evidence indicates that young adults with higher PL scores adhere to more guidelines during a 24-hour period [38]. Although our findings focus on Spanish adolescents, they align with these results. Replacing sedentary time with PA within a 24-hour period significantly enhances the health of adolescents.

Regarding our study’s findings focused on meeting the 24-hour movement guidelines, the average time spent on MVPA, sleep, and screen time by PPL status showed no significant differences between groups. These results are consistent with Liu et al. [38], where no significant interactions were found between overall PL scores and adherence to sleep duration guidelines. That study suggested that this could stem from questionnaire-based tools failing to capture these interactions effectively. Additionally, most participants in our study did not meet the 24-hour movement guidelines due to excessive screen time displayed among the sample. Globally, the use of technology among adolescents has increased significantly, leading to greater sedentary behavior and impacting various dimensions of health. Additionally, it has become a determining factor in the current mental health crisis among young people, as it replaces play-based activities with digital interactions [39]. Practical implications through PL in educational settings (emphasizing awareness and understanding of screen time use) may be crucial for fostering a balanced 24-hour routine.

In this same vein, in our current study, a total of 73.2% of adolescents met the sleep guidelines, while only 52.2% met the PA guidelines, and none of the participants adhered to the screen time guidelines. This excessive screen time may lead to numerous adverse health outcomes among young individuals. A recent global study revealed that two-thirds of adolescents spend more than two hours a day in front of screens, with some spending up to six hours daily, which reflects the results obtained from our study. This excessive exposure to screens is associated with an increased risk of metabolic syndrome, encompassing risk factors such as obesity and insulin resistance [40]. Additionally, studies have found correlations between screen time and mental health issues, including a higher prevalence of depressive symptoms and anxiety, especially when screen time replaces PA and affects sleep quality [41]. However, the review addresses how the type and use of screens impact adolescents’ mental health, emphasizing that exposure time is not the only relevant factor and that it is crucial to also consider the purpose of use. It is argued that the term ’screen time’ is less meaningful than the nature of the screen itself. Using screens for educational purposes does not pose mental health problems, whereas using social media platforms like Facebook and Instagram is associated with worse mental health outcomes and symptoms of depression and anxiety, influenced by ’fear of missing out’ (FoMO) [42].

Moreover, previous studies that investigated movement behaviors using self-report tools, such as the review by Tapia-Serrano et al. [43], have found that only 38.0% of their 1276 adolescent subjects met the PA guidelines, 82.6% met the sleep guidelines, and 15.4% met the screen time guidelines. In Spain, 34.9% of adolescents spend more than 2 h and 29.1% spend more than 3 h in front of screens. The increase in daily leisure screen time was found to be associated with shorter sleep duration [44]. Similarly, a study on Australian adolescents [41] showed that only 2.4% of 3.096 participants met all recommendations, with an average of 28.1 min of MVPA, 3.2 h of screen time, and 9.5 h of sleep per night. Additionally, in the study by Brady et al. [45], only 13% of the study population, with a mean MVPA of 33 min/day, met the guidelines for MVPA measured with an accelerometer. Hansen et al. [46] described prevalence estimates of PA, screen time, and sleep among German youths aged 9 to 18 years (n = 15,786), finding that only 9.7% of respondents met all three combined guidelines, and approximately 25% did not meet any of the guidelines. Half of the participants (50%) met the sleep guidelines, and approximately one-third met only the screen time (35%) and PA (37%) guidelines. The similarities and differences between studies can be attributed to cultural and socioeconomic factors, as well as the availability of electronic devices [47]. This highlights a global problem in which adolescents mainly do not adhere to screen time guidelines, although a good portion meets sleep guidelines and, to a lesser extent, PA guidelines. Therefore, future studies should investigate the causal relation between PPL and adherence to movement guidelines.

In analyzing PPL and adherence to 24-hour movement guidelines, our findings show that there is a significant relationship between the two, although this significance could be determined by the group that does not meet the 100% screen time adherence. On the one hand, these data align with studies conducted on children [21], where participants with higher PPL scores met the PA or sedentary behavior guidelines. However, it is important to note that, in both studies, PL was assessed using questionnaires different from those used in this study, and that the emphasis was primarily on the relation with various domains of PL. In this regard, the reported levels of PA in our study were obtained through an objective measure such as accelerometry, a gold standard method which allows for the measuring of MVPA time, which is essential for evaluating physical fitness and the level of engagement with PA, which are central aspects of PL [48]. This is in contrast with the use of self-reported questionnaires, which often have limitations due to tendencies to overestimate or underestimate PA and memory bias. On the other hand, it is evident that, as the level of PPL increases, so does the proportion of participants who meet a greater number of recommendations. Although, to our knowledge, there are no studies specifically analyzing this relationship, the results of this study can be explained by the association between greater adherence to healthy habits and higher levels of perceived physical competence and well-being [22]. However, these findings are consistent with previous studies that have shown that 56.8% of the subject adolescents met one recommendation and 22.7% met two [42]. Similarly, another study found that only 5.7% of adolescents met all three guidelines, while 9.9% met none of the guidelines [43].

Although the results obtained in this study are valuable, it is important to interpret them with caution due to certain limitations. Firstly, the participants were recruited using a convenience sampling method specifically targeting adolescents aged 12 to 16, and the sample size was small. Additionally, the use of a cross-sectional design and the small sample size in this study means that a direct cause–effect relationship cannot be established based on the findings in this study. The cross-sectional nature of the data also limits the ability to capture variations over time, which could have provided a more comprehensive view of the relationships between the studied variables. Secondly, the study took into account only the sex of the participants and not gender, limiting a series of social, cultural and psychological aspects that influence a person’s identity and behavior beyond their biological characteristics. Thirdly, the measurement of body composition using BMI may have limited predictive value for estimating body fat, bone mass and lean mass at the individual level. Fourthly, more studies are needed to objectively assess the duration and quality of sleep, as well as its relationship with screen time. Further research using different methodologies, such as interventions or longitudinal studies, is necessary to explore whether increased screen time is related to improvements in 24-hour movement behaviors among youth. Furthermore, relying on questionnaires to collect data on screen time and other variables may introduce biases, as differences in participants’ willingness to disclose information or inaccuracies in recalling details could affect the results. Response biases, such as the tendency to overestimate or underestimate certain behaviors, are common when using self-report tools and can undermine the validity of the results.

Another aspect to consider is adolescence itself, as late childhood and early adolescence cannot be strictly defined by fixed chronological boundaries. The WHO states that adolescence begins with the onset of normal puberty and ends with adult identity, approximately between ages 10 and 19 [49]. However, there is significant individual variation in the level (magnitude of change), timing (onset of change), and pace (rate of change) of biological maturation. This relative mismatch and the wide variation in biological maturation between children of the same chronological age highlight the limitations of using chronological age as a determinant. The lack of a clear consensus on classifying adolescents into specific developmental stages may also create challenges when comparing results across different studies. Finally, the findings of this study regarding sleep hours should be interpreted with caution, as the question asked refers to the time spent in bed rather than the time spent actually sleeping. It is important to consider that time spent in bed does not always reflect effective sleep time, as adolescents may spend time in bed engaging in activities unrelated to rest, such as using electronic devices. Therefore, more studies are needed to objectively assess the duration and quality of sleep and its relationship with PL levels. Additionally, longitudinal studies should be undertaken to observe how sleep patterns and PL develop over time in adolescents. By following participants for several months or years, one could identify whether changes in PL levels lead to sustainable improvements in sleep quality or if the impact varies depending on individual factors such as biological maturation or lifestyle habits. It is also important to consider specific interventions to assess whether increasing PL improves sleep. Exercise programs tailored to adolescents could be designed and their effect on sleep duration and quality monitored. Research could compare different types of exercise to determine which has the greatest impact on rest. Another relevant aspect is exploring how external factors, such as the use of electronic screens before bedtime, affect the relationship between PL and sleep. Although adolescents who engage in physical exercise may experience improvements in their sleep, exposure to blue light from electronic devices could counteract these effects. Studying these interactions would be essential for designing more effective intervention strategies.

However, this study also has several strengths. One of its main strengths lies in the objective assessment of PA levels through accelerometry, a widely accepted and validated tool in young people. The use of accelerometers allowed us to obtain more accurate and objective data on the intensity, duration, and frequency of PA among adolescents, eliminating the bias commonly associated with self-report methods such as questionnaires or diaries. Moreover, this study utilized a validated tool to assess PPL in Spanish adolescents, which reinforces the validity of the results in this specific context. Another key strength of this study is its pioneering approach in linking PPL with 24-hour movement behaviors in adolescents in Spain. To date, this association had not been established in Spanish youth, making this cross-sectional study the first to explore such an association in this population group. This may have important implications for the design of school programs, where the promotion of PA could be integrated into physical education classes, fostering the confidence, knowledge, skills, and attitudes necessary for regular physical activity throughout life. Additionally, these findings align with the goals of international organizations such as the WHO and the United Nations, and initiatives like the Global Action Plan on Physical Activity 2018–2030 and the 2030 Agenda for Sustainable Development, which prioritize the promotion of PA and physical exercise as key components for improving global health.

## 5. Conclusions

In conclusion, this is the first study to provide valuable information on the relationship between PPL levels and adherence to the 24-hour movement guidelines among Spanish adolescents. Our findings reveal that adolescents with medium and high PPL levels are more likely to meet these guidelines. It is also noteworthy that no participant met all three recommendations, with screen time being the least adhered-to category, highlighting a clear need for intervention. However, the study acknowledges the limitation of sample size, and, therefore, future research should assess the impact of PPL interventions and include a larger group of participants to achieve more representative findings. Additionally, educational programs integrating PPL as an essential component in promoting healthy habits in both educational and extracurricular contexts should be implemented.

## 6. Practical Implication

The findings of our study highlight the urgent need to implement interventions aimed at limiting screen time among adolescents through the involvement of PL. As most adolescents, regardless of their PA levels, do not meet screen time recommendations, excessive use of electronic devices, especially before bed, emerges as a widespread sedentary habit that not only negatively impacts overall sedentary time but also harms sleep quality and duration. This issue is particularly concerning in a context where access to electronic devices and social media is constant, making it difficult to control screen exposure. Educational initiatives targeting both adolescents and their families should emphasize these risks and offer concrete strategies and accessible resources to reduce screen exposure. These strategies could include scheduling “screen-free zones” at key times of the day, setting regular device use schedules, and promoting alternative non-digital activities that are appealing to adolescents, such as sports, board games, or recreational reading. These initiatives should be accompanied by informational workshops explaining the consequences of excessive screen use, addressing its impact on physical, emotional, and cognitive domains in PL. Beyond screen time, the results emphasize the need for personalized educational interventions focused on improving PA levels. Schools and community health programs play a crucial role in this area and should adopt curricula that combine traditional physical education with innovative activities that promote an understanding and appreciation of PA as an essential part of a healthy lifestyle, such as by using the personal and social responsibility model. They could also include extracurricular programs that encourage participation in sports, group walks, yoga, or dance, as well as the use of digital PA tracking tools to motivate and gamify physical exercise.

Furthermore, it was identified that some adolescents do not manage to achieve the recommended 8 h of sleep during the week, underscoring the importance of promoting healthy sleep habits. In this regard, awareness campaigns should emphasize the impact of sleep on academic performance, emotional regulation, and overall health. These campaigns could be accompanied by practical strategies, such as implementing consistent bedtime routines, reducing exposure to blue light from electronic devices at least one hour before sleep, and using relaxation techniques, such as meditation or deep breathing, to prepare the body and mind for adequate rest. Collaboration with parents and caregivers is essential, as they play a key role in establishing an environment conducive to rest and overseeing adolescents’ daily routines.

## Figures and Tables

**Table 1:** Descriptive data of participants according to perceived physical literacy status.

Participans (n)	56			
Sex				
Male (n)	40			
Female (n)	16			
Age (mean (SD)	13.2 (1.3)			
BMI (kg/m^2^) (mean (SD))	18.7 (2.5)			
**Perceived physical literacy status**				
	All	Low	Medium	High
n	56	6	9	41
**24-hour movement behaviors**				
**Physical activity**				
LPA (min/day)	259 (82.1)	246.2 (29.9)	264.4 (91.2)	258.4 (83.7)
MPA (min/day)	45.2 (21.9)	31.9 (5.6)	46.9 (18.8)	45.7 (23.3)
VPA (min/day)	29.1 (17.9)	9.9 (0.3)	30.3 (18.5)	29.9 (17.9)
MVPA (min/day)	72 (38.6)	41.7 (5.8)	77.2 (34.0)	72.6 (40.4)
Sedentary time (min/day)	515 (113.5)	514.9 (125.2)	512.4 (116.3)	515.7 (115.9)
**Sleep time**				
Weekday sleep (h/day)	8.2 (0.9)	7.4 (0.6)	8.2 (1.2)	8.3 (0.9)
Weekend sleep (h/day)	10.0 (1.2)	9.5 (1.6)	10.3 (0.9)	9.6 (1.2)
**Screen Time**				
TV (h/day)	2.3 (0.9)	2.5 (0.5)	2.4 (1.0)	2.2 (0.9)
Videogames (h/day)	2.4 (1.3)	2.2 (1.2)	2.7 (1.5)	2.4 (1.3)
Computer (h/day)	1.5 (0.8)	2.0 (1.3)	1.8 (1.1)	1.4 (0.6)
Mobile phone (h/day)	3.6 (1.4)	4.8 (0.4)	3.4 (1.1)	3.6 (1.1)
Overall screen time (h/day)	10.0 (2.6)	11.5 (1.0)	10.3 (3.5)	9.6 (2.5)

Data are presented as mean (standard deviation). According to the Spanish Perceived Physical Literacy Scale (PPL), participants were classified into three categories: low PPL (9 to 31 points), medium PPL (32 to 36 points), and high PPL (37 to 45 points). Screen time is quantified through the cut-off points of the instrument’s Likert scale (1–5). LPA = light physical activity; MPA = moderate physical activity; VPA = vigorous physical activity; MVPA = moderate-to-vigorous physical activity; Min/day = minutes per day; H/day = hours per day. Significant differences among the three PPL levels for quantitative variables were analyzed using one-way ANOVA, and Pearson’s chi-square test was used for qualitative variables (* *p* < 0.05).

## Data Availability

The datasets generated and analyzed during the current study are not publicly available due to confidentiality issues but are available from the principal investigator upon reasonable request.
